# Adventitious Root Formation in Tree Species

**DOI:** 10.3390/plants10030486

**Published:** 2021-03-05

**Authors:** Carmen Díaz-Sala

**Affiliations:** Department of Life Sciences, University of Alcalá, Alcalá de Henares, 28805 Madrid, Spain; carmen.diazsala@uah.es; Tel.: +34-91-885-5051

Adventitious root formation is a postembryonic organogenesis process induced by differentiated cells other than those specified to develop roots. Adventitious root formation is a key step in vegetative propagation by stem cuttings, and has been exploited in horticulture, agriculture, and forestry [1]. Recalcitrance to adventitious rooting from stem cuttings is a major limitation for clonal propagation, including the micropropagation of elite genotypes of many tree species [2]. For a given species, endogenous and environmental factors such as genotype, tissue and time of excision, explant position, phenology, tree age and maturation, light, temperature and the rooting conditions, such as the proliferation medium, auxin treatment, mode or time of application, rooting media or environmental conditions, affect the adventitious rooting capacity and limit the use of clonal propagation by stem cuttings or by in vitro microshoots to capture the genetic superiority of selected trees [3,4]. In addition, the need to revise protocols on an accession-by-accession basis due to the difficulty to overcome the recalcitrant behavior of some genotypes using a unique protocol has also been described [4]. Procedures for the improvement of adventitious root induction in difficult-to-root species involved not only auxin treatments, light or rooting media, but also the use of bioactive compounds that could interact with auxin activity [5]. The identification and use of new molecules enhancing the effect of auxin treatments may help improve the rooting capacity of hard-to-root species. Ricci and Rolli [5] have described the positive effect of the urea derivatives N,N0 -bis-(2,3-methylenedioxyphenyl)urea (2,3-MDPU), N,N0 -bis-(3,4-methylenedioxyphenyl)urea (3,4-MDPU), 1,3-di(benzo[d]oxazol-5-yl)urea (5-BDPU), and 1,3-di(benzo[d]oxazol-6-yl)urea (6-BDPU) on adventitious root formation in distantly related plant species. Authors report that the urea derivatives act as adventitious rooting adjuvant compounds in the presence of auxin, increasing the rooting capacity and reducing callus induction. Central to the stimulation of adventitious root development in most tree species is the application of exogenous auxin. Auxin accumulation at the rooting sites, mediated by auxin polar transport, is a specific initial response of pine rooting-competent tissues [6,7]. Urea derivatives seem to interact with local and/or long-distance auxin transport that originate auxin maxima at the rooting site or modify the sensitivity of rooting cells to auxin [5]. Auxin accumulation in the progenitor cells seems to be also crucial for xylem formation. The induction of the adventitious rooting program is antagonistic to the induction of the xylogenesis program in the rooting cambial cells [5,8,9]. Pizarro and Díaz-Sala [9] demonstrated that the rooting cells of rooting-competent pine hypocotyl cuttings may follow different morphogenic pathways, including adventitious root meristem or cambium differentiation and xylem formation, depending on the presence of exogenous auxin and the directional auxin flow. The disruption of auxin accumulation at the rooting sites in the presence of polar auxin transport inhibitors induces xylem formation and inhibits rooting. Gibberellins may act within this pathway by promoting the induction of cambial proliferation and xylem formation and by inhibiting rooting [9].

Recently, remarkable progress has been made in the mechanisms underlying adventitious rooting through the application of cutting-edge tools of genome and proteome analysis, which provide a comprehensive picture of the genes and cellular processes involved in many aspects of root induction and development, as well their interactions [10,11,12,13,14,15,16,17]. The knowledge obtained in these studies points the way forward for strategies aimed at enhancing the quantity and quality of trees for desired end-uses. Functional categories and enrichment analyses of the differentially expressed transcripts during adventitious root formation in response to auxin and other factors showed that the molecular regulation of adventitious root formation is associated with the spatio–temporal modulation of auxin-, gibberellin-, jasmonic acid- and ethylene-mediated responses, wounding and sugar signaling, sugar and amino acid metabolism, photosynthesis, cell cycle, root development pathways and genes required for meristem formation and function and stem cell identity. Justamante et al. [18] provided information for the physiological and genetic basis of adventitious rooting in carnation, using a genetic approach based on the use of an F1 collection derived from a cross between two hybrid cultivars showing contrasting rooting performances. The authors describe a negative correlation between the stem cutting area at collection time and the rooting performance, demonstrating the role of cutting itself in adventitious root formation. In addition, auxin homeostasis and ethylene synthesis are associated with the rooting behavior of different lines, concluding that both factors might also play essential roles in adventitious root formation. Duman et al. [19] have also described that the auxin–ethylene balance and cell-wall properties are associated with adventitious root formation in avocado, using transcriptomic approach based on the analysis of the differential gene expression in green and etiolated branches, as well as branches subjected to short de-etiolation Interestingly, the activation of the cambium that favors xylem differentiation may counteract the formation of adventitious roots in the green branches, which show a low rooting capacity as compared with etiolated branches or branches subjected to short de-etiolation. The mechanisms that cause a cell to maintain or lose its developmental competence are unknown. Díaz-Sala [20] has described that the expression of an array of signaling genes and a functionally complex network of molecular regulations, including epigenetic dynamics, that change during maturation to favor xylem differentiation with the loss of cell reprogramming into a root primordium could be involved in the antagonism between adventitious root formation and xylogenesis. Díaz-Sala [20] has also pointed out that the dynamic cell-wall–cytoskeleton, along with soluble factors, such as cellular signals or transcriptional regulators, may be involved in adult cell responses to intrinsic or extrinsic factors, resulting in the maintenance and the induction of root meristematic cell formation, or entrance into another differentiating pathway; which could underly recalcitrance.
